# Primary Intraosseous Meningioma of the Sphenoid-Temporal Bone with Single-Stage, 3D-Planned Cranioplasty: Case Report

**DOI:** 10.1055/a-2813-1643

**Published:** 2026-03-27

**Authors:** Arthur F. de Mattos, Mariana S. Shima, Lucas P. Chaves, João V. M. Doro, Paulo Mácio Porto de Melo

**Affiliations:** 1 Medical Department, Anhembi Morumbi School of Medicine, São Paulo, Brazil; 2 Medical Department, Universidade Municipal de São Caetano do Sul, São Paulo, Brazil; 3 Department of Neurosurgery, Hospital Militar de Área de São Paulo, São Paulo, Brazil

**Keywords:** primary intraosseous meningioma, Sphenoid-Temporal bone, osteoblastic lesion, 3D planning, custom cranioplasty

## Abstract

**Background:**

Primary intraosseous meningiomas (PIM) are rare extradural tumors, representing less than 2% of all meningiomas. They are often slow-growing and may present with nonspecific radiological features, complicating diagnosis.

**Case Report:**

We report the case of a 48-year-old female with progressive left frontotemporal bulging over 12 months, without neurological deficits. Imaging revealed an osteoblastic lesion in the left sphenoid and temporal bones, causing mild compression of adjacent structures. The patient underwent craniectomy with complete tumor resection and immediate cranioplasty using a preplanned customized prosthesis. Histopathology and immunohistochemistry confirmed a meningothelial meningioma, with tumor cells confined to intertrabecular bone spaces and free surgical margins, consistent with a primary intraosseous origin. Postoperative follow-up demonstrated excellent cosmetic results and no recurrence.

**Conclusion:**

PIMs pose diagnostic and therapeutic challenges due to their rarity and nonspecific imaging features. Histopathology remains essential for definitive diagnosis. Preoperative 3D prototyping and customized prosthesis planning can optimize surgical reconstruction, reduce deformities, and improve cosmetic and functional outcomes. This case highlights the importance of integrating clinicoradiological evaluation, histopathology, and modern reconstructive techniques to achieve complete resection and minimize recurrence.

## Introduction

Meningiomas account for approximately 36% of all primary neoplasms of the central nervous system, making them the most common primary tumor in this location. 1 The World Health Organization classification divides meningiomas into benign, atypical, or anaplastic (grades I, II, or III, respectively), according to histological features; however, other classification proposals have been suggested by different groups of specialists. 2

Although most meningiomas exhibit benign, slow-growing behavior, characterized by a well-defined intracranial lesion attached to the dura mater, these neoplasms may infiltrate adjacent structures or, in even rarer cases, originate directly in extrameningeal regions. 3 Only about 2% of all meningiomas have an extradural origin, and among these, approximately two-thirds arise directly in the cranial bones, being referred to as primary intraosseous meningiomas (PIMs). 4

In general, PIMs are benign, slow-growing lesions, but it has been suggested that there may be a relatively higher proportion of malignant transformation compared to conventional meningiomas. 5 In symptomatic cases, surgical resection combined with bone grafting constitutes the main therapeutic approach.

The understanding of this pathology as a possibility among the differential diagnoses in the presurgical complementary investigation becomes relevant in order to establish intraoperative therapeutic strategies aimed at minimizing the chances of recurrence.

This report describes a case of PIM, manifested exclusively as a progressive frontotemporal bulge.

## Illustrative Case

Female patient, 48 years old, was referred to the neurosurgery department complaining of a left frontotemporal bulge, with a hardened consistency, present for approximately 12 months, showing gradual progression over this period. The patient denied any history of trauma in the region. She reported discomfort upon local manipulation but denied spontaneous pain, visual changes, seizures, or neurological deficits. Regarding her personal history, the patient reported menopause 2 years ago, use of hormone replacement therapy with tibolone 2.5 mg daily for 1 month in 2023, previous videolaparoscopy for infertility investigation, and bilateral varicose vein surgery correction in the lower limbs. She denied other comorbidities, allergies, or substance use.

The initial clinical assessment revealed a patient who was alert and oriented to time and place, with isocoric and photoreactive pupils, preserved facial sensation, and no motor or sensory deficits, Glasgow Coma Scale 15. On inspection, a bulge of approximately 1 cm was observed in the left frontotemporal region, without signs of inflammation or superficial dermatological changes, with a hardened consistency, slightly tender to palpation, and no associated cervical, supraclavicular, or axillary lymphadenopathy.

**Fig. 1:**
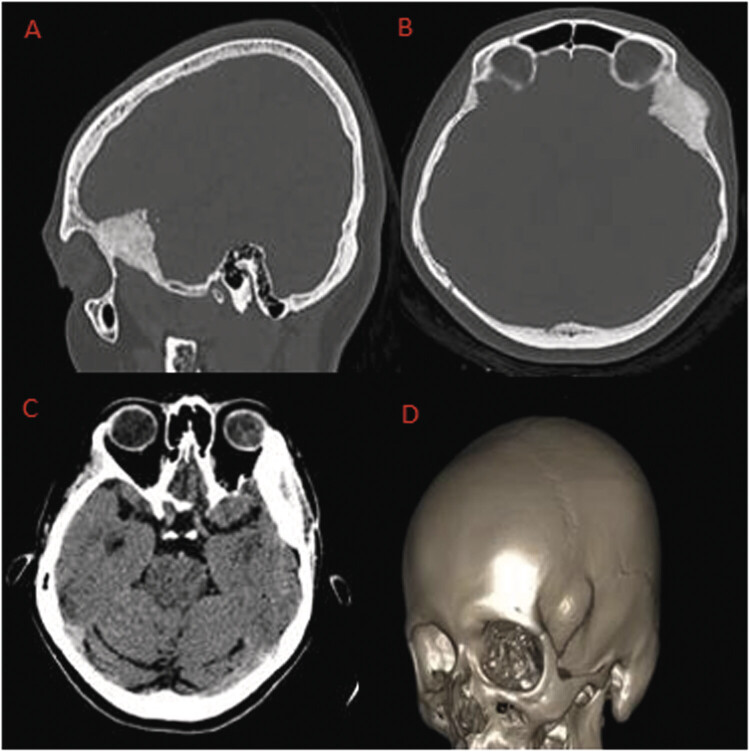
( **A** ) Bone window CT in sagittal reconstruction—expansive frontotemporal lesion with sclerosis and possible frontal bone destruction. ( **B** ) Bone window CT in axial view—expansive hyperostotic lesion in the cranial wall, consistent with an intraosseous lesion. ( **C** ) CT in axial view with windowing for brain parenchyma—slight displacement of intracranial structures adjacent to the bone lesion. ( **D** ) Three-dimensional cranial reconstruction—expansive bone lesion in the left frontotemporal region.

Given these characteristics, investigation proceeded with a computed tomography (CT) scan of the facial bones ( Fig. 1 ), which revealed an expansive bone lesion located in the left middle fossa, causing marked hyperostosis, especially in the peripterional and orbital regions, measuring 47.0 × 23.0 mm, associated with periosteal rupture and moderate periosteal reaction involving the greater wing of the sphenoid bone and the temporal bone on the left. A 3D reconstruction of the images was performed ( Fig. 1 ).

**Fig. 2:**
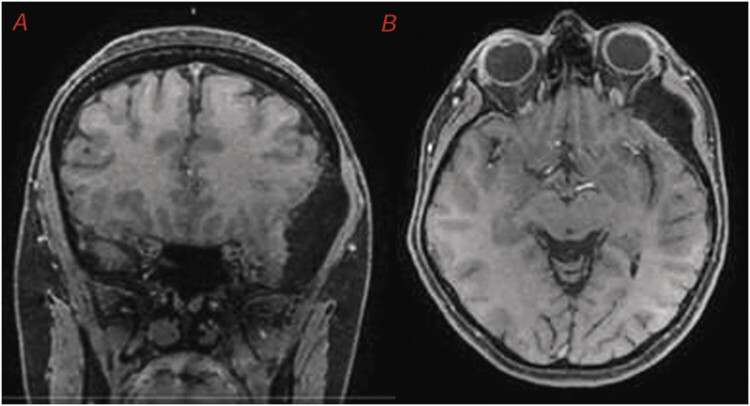
( **A** ) Coronal MRI-T1 weighted with contrast—asymmetry with deformation of the left frontal convexity. ( **B** ) Axial MRI-T1 weighted with contrast—hypodense lesion in the left frontal region suggestive of hyperostosis, consistent with an intraosseous or extra-axial lesion.

For complementary investigation, a contrast-enhanced magnetic resonance imaging (MRI) of the skull was performed ( Fig. 2 ), which demonstrated thickening and sclerosis of the left sphenoid wing, with significant asymmetry compared to the contralateral side. The lesion, measuring 40 × 21 mm, caused a mild compressive effect on the ipsilateral orbital fissure and optic canal, without signs of ocular proptosis or evident parenchymal invasion.

Given the suspicion of an intraosseous tumor with an expansive behavior and apparent compression of adjacent structures, surgical intervention was indicated. The procedure was performed via craniectomy with complete resection of the bone lesion.

Preoperatively, all radiographically abnormal bone was carefully delineated, and a craniectomy was planned to remove the entire intraosseous lesion plus an approximately 2-cm circumferential rim of normal-appearing calvaria around its radiographic margins to ensure complete and safe resection. Cranioplasty was carried out in the same surgical session using a preplanned customized prosthesis, fixed with miniplates and miniscrews.

**Fig. 3:**
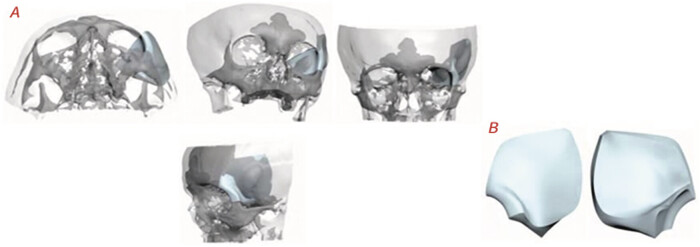
Three-dimensional cranial reconstruction simulating the prosthesis fit; prosthesis design plan.

The specimen ( Fig. 3 ) was designed following cranial anatomy, mirroring the side contralateral to the lesion. The piece was produced with a thickness of 5.00 mm in the sphenoid bone region and 2.00 mm in the internal orbital region. It was not possible to remove the affected area in the prosthesis fit simulation images for better visualization.

**Fig. 4:**
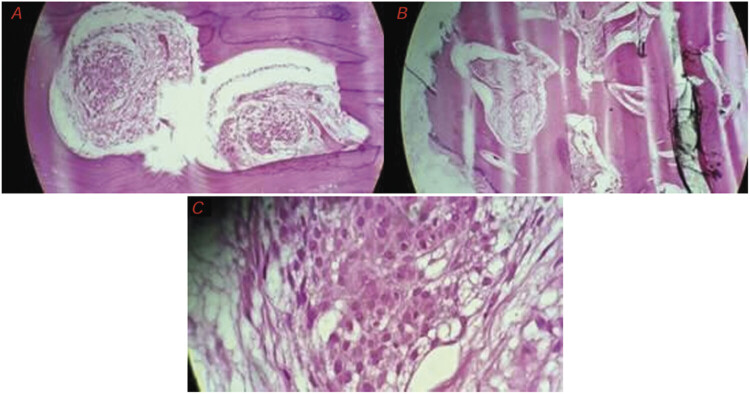
Photomicrographs of the surgically removed specimens. ( **A, B** ) Intertrabecular space infiltrated by clusters of meningothelial cells, with a whorled appearance, distributed within dense, thickened cortical bone. ( **C** ) Zooming of the tumor cells, showing a benign cytological pattern, with uniform cells, eosinophilic cytoplasm, and rounded nuclei. H&E staining.

The material collected during surgery was sent for histopathological analysis and consisted of two irregular bone fragments, originating from the greater wing of the sphenoid bone and the left temporal bone, measuring up to 27 mm × 25 mm. Microscopic examination revealed dense bone tissue with proliferation of meningothelial cells, showing well-defined cytoplasm and rounded nuclei, arranged in whorls ( Fig. 4 ). Intense hyperostosis and infiltration of the intertrabecular bone spaces were observed, without evidence of necrosis or mitotic activity. The margins were considered free.

Immunohistochemical staining showed the following profile: epithelial membrane antigen (EMA) positive, vimentin positive, progesterone receptor positive, AE1/AE3 negative, CD34 negative, and Ki-67 positive in very few cells (<2%). This immunophenotype supports the diagnosis of a benign meningothelial grade I meningioma. The most probable diagnosis was a low-grade meningioma, with extradural location and primary bone origin, strongly suggested by the absence—before, during, and after the surgical procedure—of any changes indicating adherence or direct association of the lesion with the meninges.

**Fig. 5:**
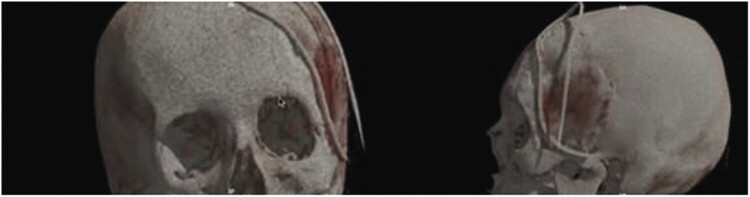
Immediate postoperative control image; 3D cranial reconstruction showing the craniectomy, prosthesis placement, and the presence of the drain inserted during the procedure.

In the immediate postoperative period, a new CT scan of the skull was performed to assess the results of the craniectomy and cranioplasty with placement of the customized prosthesis ( Fig. 5 ).

**Fig. 6:**
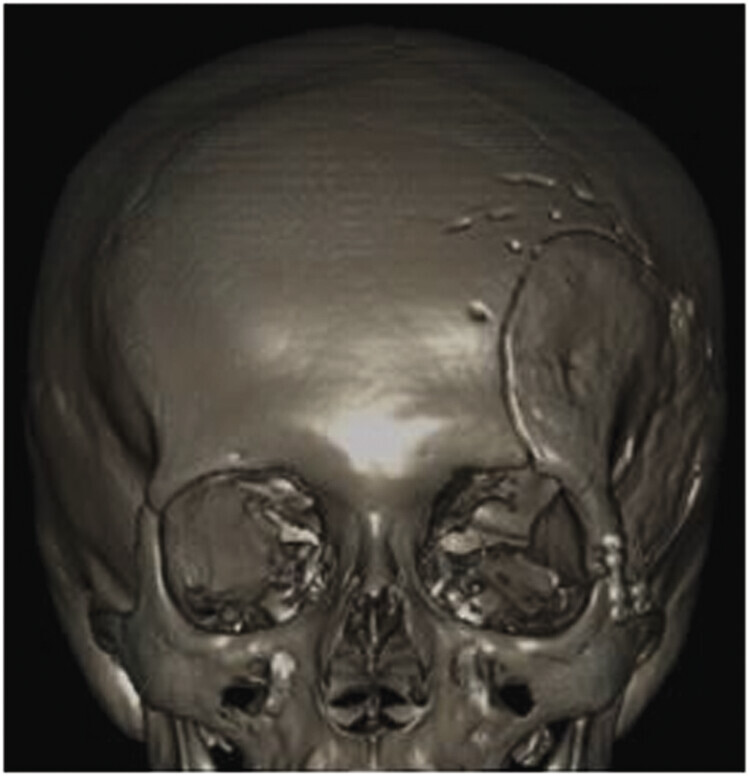
Three-dimensional cranial reconstruction—48 days after the procedure.

Forty-eight days after the procedure, a follow-up CT scan of the skull was performed, showing a good appearance of the left frontotemporoparietal cranioplasty and the customized prosthesis ( Fig. 6 ), as well as the absence of brain alterations.

**Fig. 7:**
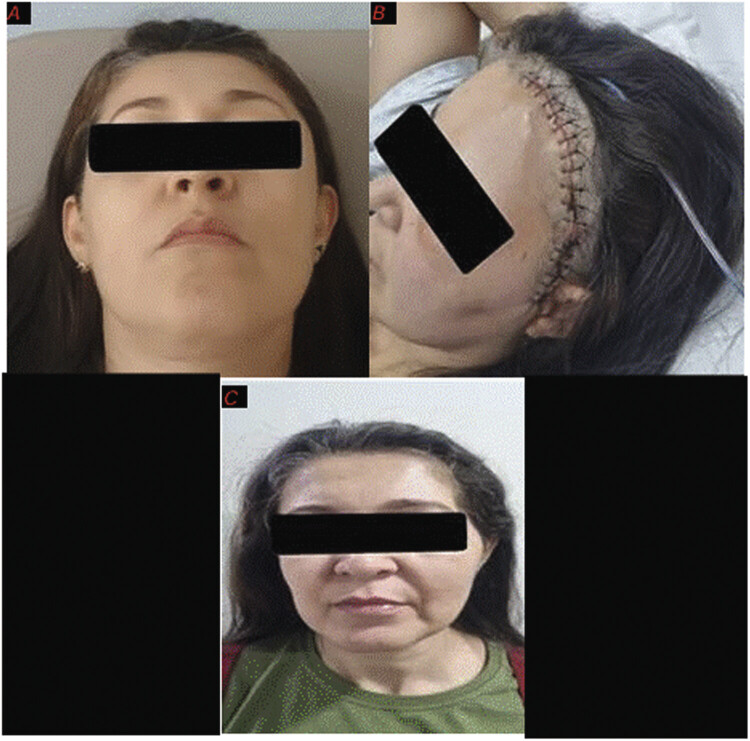
( **A** ) Patient-authorized photos in the preprocedure period, showing bulging in the left frontotemporal region. ( **B** ) Immediate postoperative period. ( **C** ) Four months after the procedure.

Compared to the preprocedure period, which showed bulging in the left frontotemporal region, postsurgery, there was normalization of the cranial appearance ( Fig. 7 ), and the patient, up to the present time, remains completely asymptomatic and without signs of lesion recurrence, satisfied with the postoperative aesthetic result.

## Discussion

PIMs are rare cranial lesions that originate directly from bone tissue. 6 The meningothelial subtype, which accounts for approximately 60% of conventional cases, is histologically characterized by the presence of uniform cells with eosinophilic cytoplasm, rounded nuclei, and whorled organization, which may undergo calcification and form psammoma bodies. 7 Among PIMs, the meningothelial subtype also stands out as the most prevalent, representing up to 47% of cases. 8 Histological analysis of the patient in the present report revealed typical features of the meningothelial subtype, with eosinophilic cytoplasm, rounded nuclei, and whorled organization.

Classically, meningiomas are well-defined intracranial masses adherent to the dura mater; however, in some cases, there is the possibility of invasion into adjacent structures, such as the cranial bones. 9 The case above, however, describes an even less common event: a meningothelial meningioma presenting as a primary bone lesion, a feature rarely associated with this type of tumor. In fact, less than 2% of all meningiomas originate outside the meninges, and PIMs account for two-thirds of these extra-meningeal tumors. 6

Most PIMs exhibit an osteoblastic pattern; however, it is estimated that around 20% of cases present as osteolytic lesions. 6 This duality gives the radiological features low specificity for the diagnosis of these tumors, making histopathological evaluation essential for their accurate identification. The radiological characteristics of the patient in the present report indicated an osteoblastic intraosseous lesion, in line with the majority of reported PIM cases.

The majority of intraosseous meningiomas are of the osteoblastic subtype, inducing hyperostosis and bone expansion. Radiologically, osteoblastic lesions typically appear as sclerotic, well-defined, and hyperdense areas, which may resemble en plaque meningioma, osteoma, osteosarcoma, Paget's disease, or fibrous dysplasia. 4

Conversely, osteolytic subtypes present as lytic and destructive bone lesions, mimicking chondroma, epidermoid cyst, osteogenic sarcoma, multiple myeloma, or metastatic disease. 10

Because of this significant radiologic overlap, establishing a preoperative diagnosis is often challenging, even in patients presenting with a slowly enlarging mass. Therefore, despite its rarity, PIM should always be considered in the differential diagnosis of both osteoblastic and osteolytic cranial lesions, as doing so can guide appropriate surgical planning.

Nevertheless, histopathologic and immunohistochemical confirmation (such as EMA and progesterone receptor positivity) remains crucial for definitive diagnosis.

In some clinical situations, distinguishing between PIMs and conventional meningiomas with bone invasion can be challenging. This differential diagnosis becomes even more complex when considering the possibility of the tumor following the reverse pathway, originating in the skull and subsequently invading the meninges.

Regarding the definition of PIM, there are divergences in the literature: some authors consider only tumors located exclusively in bone tissue as PIMs, while others also include those with primarily bony extension that show minimal dural adhesion.

In this context, Lang and colleagues proposed a classification system for primary extradural meningiomas, which can help clarify this issue. According to the classification, purely extracalvarial tumors are classified as Type I, exclusively calvarial tumors as Type II, and those with extracalvarial extension from the calvaria as Type III. Thus, PIMs can be classified as Type II or Type III, depending on the presence or absence of extracalvarial extension. 5

In our report, the most appropriate classification would be Type II, strongly suggested by the absence—on preoperative imaging and intraoperative microscopic investigation—of any changes indicating direct meningeal involvement.

There are several theories regarding the origin of PIMs. One relates to the presence of arachnoid cells trapped in the cranial sutures, a phenomenon that could occur during the cranial molding process in the neonatal period. Another hypothesis suggests that meningothelial cells could be incorporated into areas of bone fractures as a result of cranial trauma. 11 However, studies based on case series do not consistently support these theories. An analysis compiling 168 reports of calvarial meningiomas revealed that only a small portion—about 8%—was associated with cranial sutures. Another review, involving 36 cases, highlighted that only 14% had a history of trauma in the affected region. 5

In our case, the tumor appeared to originate near the spheno-frontal and spheno-squamosal sutures, and the patient had no history of trauma in the region. An alternative explanation involves the anomalous differentiation of multipotent mesenchymal stem cells present in the bone region, which could differentiate into arachnoid cells directly within the intraosseous area. 12

The bony regions of the orbit and the frontoparietal calvaria are the most common sites for PIM occurrence. 13 However, these tumors can theoretically arise in any cranial area. In our case, the lesion was located in the frontotemporal region, similar to what is most commonly described in the literature.

In addition to bulging, meningiomas can cause other signs and symptoms, such as headache, focal neurological deficits, obstructive hydrocephalus, proptosis, and seizures. 1 However, the patient in this report presented exclusively with the complaint of bulging, without any neurological signs or symptoms.

Primary intracranial meningiomas have a higher malignancy profile (11%) compared to intradural meningiomas (2%), although they are usually also benign and slow-growing. 10 According to the histopathological analysis of the reported case, the finding of a neoplasm with free margins and absence of mitoses confirmed its benign nature; the uniform meningothelial cells arranged in whorls characterize this case as a grade I meningothelial meningioma. However, the meningothelial cells were located exclusively within the intertrabecular bone regions, indicating, due to the complete absence of dural contact with the tumor, a primary intraosseous origin.

Immunohistochemistry is a valuable adjunct in confirming the diagnosis of PIM and differentiating it from other osteogenic or mesenchymal lesions. In our case, positivity for EMA, vimentin, and progesterone receptor, along with negativity for AE1/AE3 and CD34, established the meningothelial nature of the lesion and helped to exclude other diagnoses. A low Ki-67 proliferation index further supports the benign biological behavior, typical of World Health Organization grade I meningiomas.

The combined radiologic, histopathologic, and immunohistochemical findings were indicative of a primary intraosseous meningothelial meningioma, excluding alternative diagnoses such as fibrous dysplasia, osteoma, or metastatic lesions.

Surgical intervention was indicated due to suspicion of an expansile bone tumor. The approach consisted of a cranioplasty with placement of a preplanned customized prosthesis. Preoperative prototyping is a strategy that uses 3D reconstruction of the cranial CT scan, allowing for prior simulation and evaluation of incision and prosthesis fixation sites, while comparing the symmetry of the reconstruction with the healthy side. In our experience, this type of planning helps reduce surgical time and minimize bone deformities, which are often the main challenge in cases of deforming expansile tumors.

Somatostatin receptor–based positron emission tomography (PET) imaging, such as [68-Gallium]-DOTATATE PET/CT, has emerged as a modality for detecting meningiomas due to their strong expression of somatostatin receptors 2 (SSTR2), and may be particularly useful in patients with atypical presentations or equivocal conventional imaging. 14 In the present case, however, the lesion was solitary and radiographically well circumscribed on CT and MRI, and additional functional imaging was not obtained, as it was unlikely to modify the surgical plan.

The prosthesis was digitally modeled using a 3D reconstruction of thin-slice CT data. Virtual mirroring of the contralateral skull served as a template to achieve anatomical symmetry. The customized implant, fabricated from polyetheretherketone, was sterilized and intraoperatively adjusted for precise contour alignment with the adjacent bone edges. This approach minimized dead space, ensured optimal fixation with miniplates, and produced excellent aesthetic restoration.

Wide surgical resection is the main treatment for extradural meningiomas and can be curative when complete tumor removal is achieved. In our report, total tumor excision was accomplished, as confirmed by histopathological analysis of free margins. The patient experienced an excellent recovery, remains completely asymptomatic, shows no evidence of recurrence to date, and reports full satisfaction with the postoperative aesthetic outcome.

However, the report also has limitations. As with most case reports, generalizability is restricted, and conclusions about prognosis or optimal management cannot be drawn from a single patient. Additionally, while the cosmetic and clinical results are satisfactory, the relatively short follow-up period may not fully exclude the risk of late recurrence, which may occur even years after resection.

## Conclusion

PIM should remain in the differential diagnosis of cranial bone lesions—even when symptoms are minimal—because radiologic findings are often nonspecific. Early tissue confirmation (including intraoperative frozen section when available) can refine the operative plan in real time. Management hinges on clinicoradiologic–pathologic correlation to distinguish PIM from mimics and to define resection margins. When feasible, preoperative 3D planning with a patient-specific implant enables a single-stage workflow—definitive resection plus precise reconstruction—supporting efficient surgery and excellent functional and cosmetic outcomes.
